# Exploring the role of apolipoprotein E gene promoter polymorphisms in susceptibility to normal-tension glaucoma in a Korean population

**DOI:** 10.1038/s41598-024-58442-8

**Published:** 2024-04-18

**Authors:** Young Jun Park, Hye-Young Shin, Jong-Il Kim

**Affiliations:** 1 Medical Research Center, Genomic Medicine Institute, Seoul National University, 101 Daehak-ro, Jongno-gu, Seoul, Republic of Korea; 2 Department of Ophthalmology, Uijeongbu St. Mary’s Hospital, College of Medicine, The Catholic University of Korea, 271, Cheonbo-ro, Uijeongbu-si, Gyeonggi-do Republic of Korea; 3 Department of Ophthalmology, Uijeongbu St. Mary’s Hospital, College of Medicine, The Catholic University of Korea, Seoul, Republic of Korea; 4 Department of Biochemistry and Molecular Biology, Seoul National University College of Medicine, Seoul, Republic of Korea; 5 Department of Biomedical Sciences, Seoul National University College of Medicine, Seoul, Republic of Korea

**Keywords:** Haplotypes, Glaucoma

## Abstract

Glaucoma, particularly primary open-angle glaucoma (POAG), poses a significant global health concern. Distinguished by intraocular pressure (IOP), POAG encompasses high-tension glaucoma (HTG) and normal-tension glaucoma (NTG). Apolipoprotein E (APOE) is a multifaceted protein with roles in lipid metabolism, neurobiology, and neurodegenerative diseases. However, controversies persist regarding the impact of *APOE* single-nucleotide polymorphisms (SNPs) on open-angle glaucoma and NTG. This study aimed to identify *APOE*-specific SNPs influencing NTG risk. A cohort of 178 patients with NTG recruited from Uijeongbu St. Mary’s Hospital and 32,858 individuals from the Korean Genome and Epidemiology Study (KoGES) cohort were included in the analysis. Genotype and haplotype analyses were performed on three promoter SNPs (rs449647, rs769446, and rs405509) and two exonic SNPs (rs429358 and rs7412) located on chromosome 19. Among the five SNPs, rs769446 genotypes exhibited significant differences between cases and controls. The minor allele C of rs769446 emerged as a protective factor against NTG. Furthermore, haplotype analysis of the five SNPs revealed that the A-T-G-T-T haplotype was a statistically significant risk factor for NTG. This study indicated an association between *APOE* promoter SNPs and NTG in the Korean population. Further studies are required to understand how *APOE* promoter SNPs contribute to NTG pathogenesis.

## Introduction

Glaucoma is a chronic, progressive disease that irreversibly damages the optic nerve, potentially leading to blindness^1^. Among the various types, primary open-angle glaucoma (POAG) is the most prevalent globally, with notable variations in prevalence among different racial groups^2^. POAG is further categorized into high-tension glaucoma (HTG) and normal-tension glaucoma (NTG) based on intraocular pressure (IOP). Unlike HTG, where IOP exceeds 22 mmHg, NTG is characterized by glaucomatous optic nerve damage despite having IOP within the normal range. In Korea, consistent with patterns in other East Asian populations, NTG is the most common type of glaucoma^3^. The Korea Namil study reported that patients with NTG comprised 77% of patients with POAG in Central South Korea^3^. Notably, open-angle glaucoma (OAG), including NTG, exhibits considerable phenotypic and genetic diversity and is predominantly polygenic in its genetic architecture^4,5^.

Apolipoprotein E (APOE) is a versatile protein that plays crucial roles in lipid metabolism, neurobiology, and neurodegenerative diseases^6–8^. The *APOE* gene, located on chromosome 19, gives rise to three major isoforms—apoE2, apoE3, and apoE4—encoded by the ε2, ε3, and ε4 alleles, respectively, formed by haplotype combinations of exonic SNPs (rs429358 and rs7412)^9^. This gene has been extensively studied in Alzheimer’s and Parkinson’s diseases, where the ε4 allele is a prominent risk factor for late-onset Alzheimer’s disease (AD)^9,10^, and its association with glaucoma susceptibility has been explored in diverse populations^11–23^. In addition to the *APOE* gene ε2/ε3/ε4 polymorphism, investigations have delved into polymorphisms at the *APOE* gene promoter region (rs449647, rs769446, and rs405509 SNPs) in relation to glaucoma^13,16,21–23^. However, previous studies have reported controversial results concerning the association between POAG and *APOE* gene polymorphisms, potentially stemming from discrepancies in study group designs, diagnostic criteria/subtypes, and insufficient statistical power across studies.

To the best of our knowledge, the genetic association between multiple *APOE* SNPs and glaucoma has not been previously explored within the Korean population. Therefore, the present study aims to address this gap by investigating the association between *APOE* polymorphisms and NTG and to identify *APOE* haplotypes that may influence NTG susceptibility in the population of the Republic of Korea.

## Methods

### Participants

One hundred and seventy-eight unrelated patients with NTG were recruited from the glaucoma clinic of Uijeongbu St. Mary’s Hospital, Korea. NTG was defined based on an open angle observed through gonioscopy, characteristic optic disk changes (i.e., neuroretinal rim thinning, notching, and/or an RNFL defect), and corresponding visual field (VF) defects. Confirmation required at least two VF tests in at least one eye, and an IOP of less than 22 mmHg. Patients with angle-closure glaucoma, secondary glaucoma resulting from uveitis or neovascularization, pseudoexfoliation, or a history of ocular trauma or steroid treatment were excluded from the study. All participants signed informed consent forms prior to enrollment after being briefed on the purpose of the genetic testing process. Patients who refused to participate were excluded, and the study received approval from the Institutional Review Board of Uijeongbu St. Mary’s Hospital (IRB No. UC20SISI0142), in which all aspects were in accordance with the principles of the Declaration of Helsinki. The case group comprised 178 patients with an average age of 64.9 (Standard deviation 12.2), including 65 male (36.5%) and 113 female (63.5%) patients.

For haplotype analysis, a control group comprising 32,858 individuals without self-reported glaucoma from the Korean Genome and Epidemiology Study (KoGES) cohort was utilized. KoGES is a large prospective cohort study designed to identify gene and environment factors and their interactions in common complex diseases, such as metabolic syndrome and cancer. All ethical considerations and requirements were met, and the need for informed consent was waived owing to complete participant anonymity. Among the KoGES cohort, the Health examinee cohort, or the city cohort, comprising 58,700 people, was utilized. Among these, 32,858 participants self-reported in a questionnaire that they had no history of glaucoma until the day of screening. IRB approval was obtained and confirmed by the Center for Disease Control of the Korean government, and the execution of this study was approved by the Seoul National University Hospital Clinical Research Institute (IRB No. C-2004-080-1117). Similarly, these specific methodologies of this study were implemented adhering to the principles of the Declaration of Helsinki. The control group comprised 32,858 participants with an average age of 58.0 (Standard deviation 5.6), including 11,846 male (36.1%) and 21,012 female (63.9%) participants. The control group was filtered to include individuals aged between 50 and 79 years, ensuring a direct and relevant comparison with the case group.

### Genotyping and imputation

**Figure 1:**
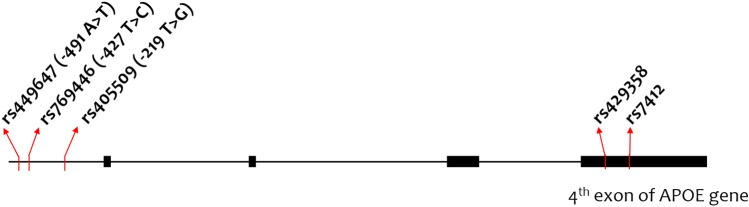
Gene structure of single-nucleotide polymorphisms (SNPs) in the apolipoprotein E (*APOE*) gene locus.

We selected five SNPs in the *APOE* gene, including three promoter SNPs (rs449647, rs769446, and rs405509) and two exonic SNPs (rs429358 and rs7412) (Fig. 1).

In the case group, genotyping for these five SNPs was performed using the TaqMan SNP Genotyping Assay on the QuantStudio Dx Real-Time PCR instrument (Applied Biosystems, Foster City, CA, USA).

For the control group, samples were genotyped using the Affymetrix Axiom Korean Chip (Version 1.1) and imputed with the Northeast Asian Reference Database panel. A total of 7,857,085 variants meeting criteria (minor allele frequency ≥ 0.01, Hardy–Weinberg equilibrium *p*-value ≥ 1E-6, missing rate < 0.1, and estimated imputation accuracy from Minimac4 (R^2^) ≥ 0.3) were used for analyses. The genotypes were extracted using PLINK software (Version 1.9)^24^, and all other statistical analyses were conducted using STATA (Version 17.0; StataCorp. LLC, College Station, TX, USA).

The minor allele frequencies (MAFs) for each *APOE* SNP are presented in Table 1, with data sourced from the African, American, European, South Asian, East Asian, and Korean Reference Genome Databases (KRGDB; N = 2,922 participants).

**Table 1:** General information and MAF from 1000 Genomes database of five investigated SNPs of the *APOE* gene.

SNP	Genotype	Position	1000 genome database (MAF)						KRGDB
	(Major/Minor)	(GRCh38)	All	AFR	AMR	EUR	SAS	EAS	(MAF)
rs449647	A/T	chr19:44,905,307	0.1997	0.3321	0.258	0.165	0.193	0.0268	0.0359
rs769446	T/C	chr19:44,905,371	0.0647	0.034	0.036	0.0785	0.071	0.1052	0.0746
rs405509	T/G	chr19:44,905,579	0.5282	0.7632	0.497	0.5159	0.446	0.333	0.2969
rs429358	T/C	chr19:44,908,684	0.1506	0.2678	0.104	0.1551	0.087	0.0863	0.0917
rs7412	C/T	chr19:44,908,822	0.0751	0.1029	0.048	0.0626	0.044	0.1002	0.0693

*MAF* minor allele frequencies; *SNP* single-nucleotide polymorphism; *APOE* apolipoprotein E; All, average of the entire population; *AFR* African, *AMR* American; *EAS* East Asian; *EUR* European; *SAS* South Asian; *KRGDB* Korean Reference Genome Database.

APOE reference sequence is NP_001289619.1 (isoform b precursor).

### Statistical analysis

Allele and genotype frequencies in cases and controls were compared using *p*-values derived from chi-square or Fisher’s exact tests.

Haplotype analysis was performed using SHE-sis web-based software^25,26^ on five *APOE* SNPs located on chromosome 19 (Fig. 1). For this particular analysis, frequencies below 0.01 in both the control and case groups were excluded.

Statistical significance was set at *p* < 0.05, and all genomic computational analyses were performed on the computing server at the Genomic Medicine Institute Research Service Center. Bonferroni corrected *p*-value applied for the analysis involving the 5 SNPs, with the significance threshold set at *p* < 0.05/5 = 0.01, and for the haplotype analysis, with the significance threshold set at *p* < 0.05/7 = 0.007, which accounts for 7 haplotypes.

## Results

General information and MAF of the five SNPs from the 1000 Genomes Project database are shown in Table 1. Because our study population consisted of South Koreans, MAF values closely resembled those in the KRGDB but exhibited significant deviations from East Asians in the 1000 Genomes Project, particularly for rs769446, rs405509, and rs7412, considering the sample sizes (East Asian MAF, all *p* < 0.05; KRGDB MAF, *p* = 0.3, 0.19, and 0.41, respectively, compared to our study).

In Table 2, allele counts, allele frequencies, genotype counts, and genotype frequencies for *APOE* 3 promoter SNPs (rs449647, rs769446, and rs405509) and two exonic SNPs (rs429358 and rs7412) are detailed for patients with NTG and controls. Among the five SNPs, only the second promoter (rs769446) was significantly different between the case and control groups (allele frequency difference, *p* = 0.008; genotype frequency difference, *p* = 0.009, reaching the Bonferroni corrected *p*-value thresholds of 0.01). The minor allele C of rs769446 was identified as a protective allele against NTG. However, rs449647, rs405509, rs429358, and rs7412 allele and genotype frequencies were not significantly different between cases and controls.

**Table 2:** Genotype and allele frequencies of the promoter region and exon 4 SNPs of the *APOE* gene.

Polymorphism	Allele count (Allele Frequency, %)				Genotype count (Genotype frequency, %)			
	Allele	NTG, N = 356	Control, N = 65,716	*p*-value	Genotype	NTG, N = 178	Control, N = 32,858	*p*-value
rs449647	A	345 (96.9)	63,752 (97.0)	0.91	AA	168 (94.6)	30,922 (94.1)	0.09
	T	11 (3.1)	1964 (3.0)		AT	9 (4.9)	1908 (5.8)	
	OR (T)	1.03			TT	1 (0.5)	28 (0.1)	
rs769446	T	344 (96.6)	61,128 (93.0)	**0.008**	TT	166 (93.3)	28,429 (86.5)	0.009*
	C	12 (3.4)	4588 (7.0)		TC	12 (6.7)	4270 (13.0)	
	OR (C)	0.46			CC	0 (0)	159 (0.5)	
rs405509	T	268 (75.3)	46,931 (71.4)	0.11	TT	102 (57.3)	16,801 (51.1)	0.25
	G	88 (24.7)	18,785 (28.6)		TG	64 (36.0)	13,329 (40.6)	
	OR (G)	0.82			GG	12 (6.7)	2728 (8.3)	
rs429358	T	323 (90.7)	59,679 (90.8)	0.96	TT	147 (82.6)	27,117 (82.5)	0.95
	C	33 (9.3)	6037 (9.2)		TC	29 (16.3)	5445 (16.6)	
	OR (C)	1.01			CC	2 (1.1)	296 (0.9)	
rs7412	C	341 (95.8)	61,421 (93.5)	0.08	CC	163 (91.6)	28,704 (87.4)	0.09†
	T	15 (4.2)	4295 (6.5)		CT	15 (8.4)	4013 (12.2)	
	OR (T)	0.63			TT	0 (0)	141 (0.4)	
*APOE* alleles	ε2	15 (4.2)	4295 (6.5)		ε2/ε2	0 (0)	141 (0.4)	
	ε3	308 (86.5)	55,384 (84.3)		ε2/ε3	14 (7.9)	3650 (11.1)	
	ε4	33 (9.3)	6037 (9.2)		ε3/ε3	133 (74.7)	23,326 (71.0)	
					ε3/ε4	28 (15.7)	5082 (15.5)	
					ε2/ε4	1 (0.6)	363 (1.1)	
					ε4/ε4	2 (1.1)	296 (0.9)	

Significant values are in bold.

*SNP* single-nucleotide polymorphism; *APOE* apolipoprotein E; *NTG* normal tension glaucoma; *OR* odds ratio; Parentheses indicate the effect allele that was used for the calculation of odds ratios.

*Comparison of the two groups: TT versus TC + CC.

^†^Comparison of the two groups: CC versus CT + TT.

The distribution of the *APOE* ε2/ε3/ε4 genotypes and alleles in both NTG and control groups are also shown in Table 2. The ε3 allele exhibited the highest percentage among cases and controls, followed by the ε4 allele, with the ε2 allele showing the lowest percentage. There were no significant differences in allele frequency and genotype frequencies regarding the ε2/ε3/ε4 alleles. Additional analyses, comparing ε2 allele carriers (ε2/ε2, ε2/ε3, ε2/ε4 genotype population) versus all non-carriers of the ε2 allele (*p* = 0.09, chi-squared), ε4 allele carriers (ε4/ε4, ε3/ε4, ε2/ε4 genotype population) versus non-carriers of the ε4 allele (*p* = 0.98, chi-squared), and ε4/ε4 versus non-ε4/ε4 subgroups (*p* = 0.68, Fisher’s exact test) did not reveal statistically significant differences.

Haplotype results for the five *APOE* SNPs are presented in Table 3. Notably, the A-T-G-T-T haplotype (risk, *p* = 4E-05, odds ratio 6.252, 95% confidence interval: 2.299–17.003) reached Bonferroni-corrected statistical significance. This particular haplotype was associated with the *APOE* ε2 haplotype (rs429358, rs7412: T and T, respectively). However, the *APOE* ε3 and ε4 haplotypes (rs429358, rs7412: T and C, C and C, respectively) did not exhibit significance in haplotype analysis (Table 3).

**Table 3:** Haplotype frequencies of the five SNPs of the NTG and control groups.

Haplotype	APOE status	NTG case (freq)	Control (freq)	*p*-value	Odds ratio	95% CI
ACGTT	ε2	0.031	0.063	0.01121	0.467	[0.256–0.853]
**ATGTT**	ε2	0.011	0.002	**0.00004**	6.252	[2.299–17.003]
ATGTC	ε3	0.19	0.206	0.43887	0.901	[0.691–1.174]
ATTTC	ε3	0.66	0.619	0.14767	1.177	[0.944–1.467]
TTGTC	ε3	0.012	0.011	0.83992	1.105	[0.420–2.902]
ATTCC	ε4	0.073	0.072	0.96167	1.01	[0.677–1.505]
TTTCC	ε4	0.019	0.018	0.87286	1.064	[0.497–2.277]

Significant values are in bold.

*SNP* single-nucleotide polymorphism; *APOE* apolipoprotein E; *NTG* normal-tension glaucoma; *freq* frequency; *CI* confidence interval.

The five single-nucleotide polymorphisms used for haplotype analysis are ordered as follows: rs449647, rs769446, rs405509, rs429358, and rs7412.

Frequency < 0.01 in both control and case has been dropped.

Bonferroni-corrected *p*-value was applied as 0.05/7 = 0.007.

## Discussion

Although the genetic link between Alzheimer’s disease and glaucoma etiology remains unclear, recent studies have focused on identifying shared genetic risk factors between AD and glaucoma, which are neurodegenerative diseases whose prevalence increases with age^27,28^. The relationship between polymorphisms in the *APOE* gene and POAG has been addressed in many case–control studies^11–23,29^. However, these studies have been conducted on different glaucoma subtypes and in different ethnic groups, and the reported results are inconsistent^30–33^. Therefore, in this study, we investigated the genetic association between *APOE* gene polymorphisms and NTG in the Korean population. Contrary to expectations, we identified an inverse association between NTG and a specific *APOE* promoter SNP (rs769446 C allele), positively associated with AD. Additionally, in our haplotype analysis involving the five SNPs, we have identified the A-T-G-T-T haplotype as a risk factor for NTG.

The present study revealed no association between NTG and *APOE* ε2/ε3/ε4 alleles in the Korean population. Conflicting studies have been previously published regarding the relationship between *APOE* ε2/ε3/ε4 alleles and glaucoma^11–20^, with mixed results from meta-analyses. While some studies associate *APOE* ε2/ε3/ε4 alleles with glaucoma^33,34^, others report no such association^31,32^. Two studies reported an association between OAG and *APOE* gene polymorphisms in Asians^33,34^, whereas other studies included in the meta-analysis patients with both HTG and NTG. Therefore, no conclusions can be drawn regarding the association between *APOE* and NTG. Individual studies on NTG subtypes have also reported conflicting results. While Lam et al. and Margeta et al. suggested a protective association between the *APOE* ε4 allele with NTG^16,35^, Vicker et al. indicated that the *APOE* ε4 allele was associated with elevated risk for glaucoma in the Tasmanian population^20^. Additionally, Lake et al. reported no association between the ε4 allele and NTG in Caucasians^18^. The discrepancies in previous study results may be attributed to the wide geographical variability in *APOE* ε4 distribution, as has been well-documented previously^7^. Therefore, although we identified no association between NTG and the *APOE* ε2/ε3/ε4 alleles in the Korean population, it is difficult to rule out the possibility that results in other ethnic groups may differ from our findings.

The rs449647, rs769446, and rs405509 SNPs are located in the promoter region of *APOE*. In a meta-analysis on the association between the promoter of the *APOE* gene and glaucoma, rs449647 was reported to be associated with HTG but not with NTG^30^. However, another meta-analysis by Guo et al. reported that promoter SNPs of the *APOE* gene (rs449647, rs769446, and rs405509 SNPs) were not associated with POAG^23^. Contrary to the results reported by Guo et al., our study results indicated that among the three promoter SNPs, the rs769446 C allele was significantly associated with NTG. These discrepancies may stem from variations in glaucoma subtypes. In our study, we also revealed that the rs449647 and rs405509 SNPs did not show any association with NTG, consistent with the study by Chen et al.^30^ and contrary to that by Guo et al.^23^. The difference in the results of other previously reported individual studies can be attributed to the inclusion of not only different glaucoma subtypes but also different ethnic populations. rs769446 minor allele (C) was more common in East Asians (10.5%) than in Americans (3.40%) or Africans (3.60%) (1000 Genomes Project, Table 1). The MAF of the rs769446 minor allele (C) of KRGDB was 7.46%, with our control group exhibiting a MAF of 7.0%. Furthermore, the five allele frequencies of *APOE* polymorphisms in the 1000 Genomes Project exhibited significant differences between different ethnic groups (Table 1). Therefore, caution is warranted in interpretation, considering the focus of our study on Korean patients with NTG. Moreover, in the present study, we selected three promoter SNPs, given their extensive documentation in previous studies regarding apolipoprotein E, as stated above. We selected the two exonic SNPs (rs429358 and rs7412) because they are the sole determinants for the *APOE* ε2/ε3/ε4 alleles^36^. However, haplotype analysis studies exploring the combination of these five SNPs are relatively scarce in the current literature.

Among all SNPs, only the C allele of rs769446 was significantly associated with NTG (*p* = 0.008, Table 2) in the present study. Promoter polymorphisms of the *APOE* gene have been linked to increased plasma APOE levels in patients with AD and an elevated risk of AD^37–42^. These associations are attributed to the likelihood that the promoter SNP induces changes in the transcriptional activity of the gene^38^. Previous research has demonstrated that rs769446 is a functional polymorphism, exhibiting an association with plasma APOE concentration regardless of ε2/ε3/ε4 genotypes^43^. Additionally, a recent meta-analysis of promoter SNPs of the *APOE* gene reported that the rs769446 SNP was associated with an increased risk of AD^37^. Although NTG and AD have different pathogenesis, and the mechanism by which *APOE* influences the risk of two diseases may be different, our study results are supported by the fact that functional promoter SNPs (rs769446) were independently associated with specific diseases regardless of *APOE* exonic SNPs.

In the present study, haplotype frequency estimation exhibited a significantly increased frequency of the A-T-G-T-T haplotype in patients with NTG (*p* < 0.001, Table 3). Therefore, we suggest that the rs769446 T allele acts as a genetic risk factor specifically within the ε2 haplotype (T-T), but not in the more prevalent ε3(T-C) or ε4(C–C) *APOE* haplotype. Our results provide evidence that *APOE* promoter polymorphisms modulate the risk of NTG development within the Korean population. These effects of this haplotype may influence risk stratification for glaucoma.

Our study has several limitations. First, we selected only three SNPs in the promoter region and two exonic SNPs of *APOE* based on previous studies. However, in terms of allele frequencies, all five SNPs had a MAF of over 0.01, and according to the KRGDB, four of these SNPs have a MAF exceeding 0.05, which further justified the criteria for the selection of common SNPs for haplotype analysis. Second, we could not investigate the association between these significant polymorphisms and NTG severity because NTG genetic data were not sufficiently available. In the future, the association between these SNPs/haplotypes and clinical features, such as glaucoma severity, should be investigated in a large NTG cohort. Third, the KoGES control population was not ophthalmologically screened for glaucoma. However, subject misclassification within the control group was expected to bias the analyses toward the null hypothesis. Fourth, as shown in Table 1, the five SNPs of *APOE* included in this study showed significant differences in MAF across different ethnic groups. Therefore, multicenter, well-designed studies with larger sample sizes and ethnic groups are required to validate these results. Finally, the case and control groups were not matched with propensity scores in our study. Nevertheless, the age distribution and sex proportions were similar and fell within the acceptable range for statistical analysis. Further, because NTG is more prevalent in the older population, the case group had an older age mean value (64.9 vs. 58.0) compared to the control group, as expected. Therefore, some of the findings reported in our study should be interpreted with caution in light of these limitations.

In summary, we reported, for the first time, an association between *APOE* promoter SNPs and NTG in the Korean population. Our results clarify the overall contribution of *APOE* gene polymorphisms to the risk of developing NTG in the Korean population. Further studies using animal glaucoma models are needed to understand the mechanism by which *APOE* promoter SNPs are involved in NTG pathogenesis.

## Data Availability

The KoGES genotype and phenotype data used for this study are made available to researchers after submitting an appropriate research proposal with applicable Institutional Review Board consent and completing the quality control process of the National Research Institute of Health of Korea. More information regarding this database can be found at www.kdca.go.kr (Korea Disease Control and Prevention Agency) website.

## Abbreviations

AD: Alzheimer’s disease
APOE: Apolipoprotein E
IOP: Intraocular pressure
KoGES: Korean Genome and Epidemiology Study
KRGDB: Korean Reference Genome Database
MAF: Minor allele frequency
NTG: Normal-tension glaucoma
OAG: Open-angle glaucoma
POAG: Primary open-angle glaucoma
SNP: Single-nucleotide polymorphism
